# On the functional redundancy of alcohol tolerance in rice societies

**DOI:** 10.1017/ehs.2026.10050

**Published:** 2026-04-23

**Authors:** Berthold Wigger

**Affiliations:** Chair of Public Finance and Public Management, Karlsruhe Institute of Technology, Karlsruhe, Germany

**Keywords:** ALDH2 mutation, functional redundancy, Rice Theory of Culture, gene–culture coevolution

## Abstract

This article analyses the evolutionary genesis of the aldehyde dehydrogenase 2 mutation in East Asia through the lenses of institutional economics. While biology typically frames the absence of alcohol tolerance as a metabolic defect, this paper proposes the concept of functional redundancy. We argue that the specific social organization of rice societies – characterized by deep material interdependence – rendered alcohol consumption superfluous as an instrument for trust-building and social cohesion. The resulting genetic path dependency illustrates how historical institutional frameworks continue to shape the biological constitution of modern populations.

## Graphical abstract

**Figure:**
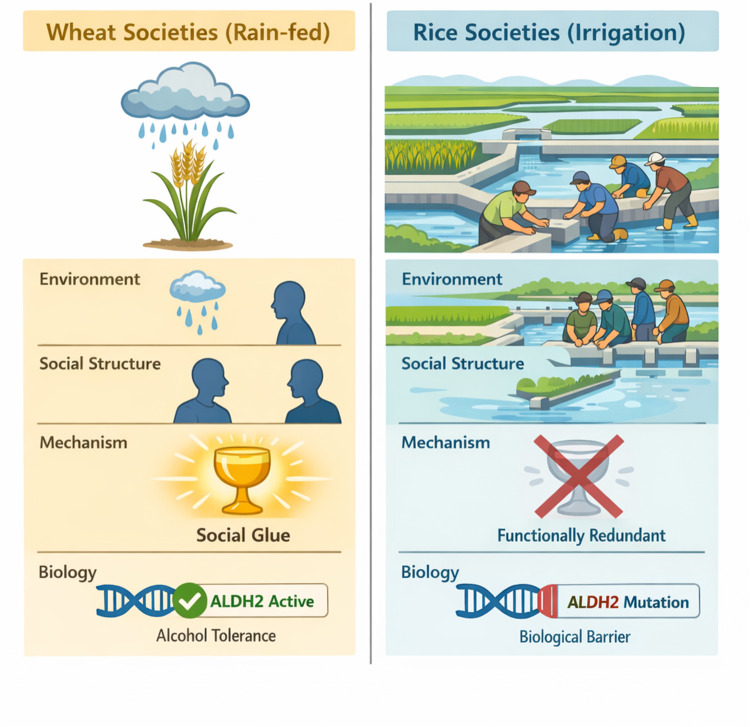

## Social media summary

Rice cultivation made alcohol’s social role redundant, leading to alcohol intolerance.

## 1. Introduction

In the social sciences, human biology is frequently treated as a constant background variable. However, modern research into gene–culture coevolution suggests that the genetic make-up of a population can be understood as the result of millennia of adaptation to specific socioeconomic niches. A prominent example of this process is the East Asian aldehyde dehydrogenase 2 (ALDH2) mutation, which leads to a massive alcohol intolerance in nearly half of the affected population. The geographic correlation of this mutation with the centres of early paddy rice cultivation in the Yangtze Basin (Li et al., 2009) suggests the need for an interdisciplinary approach. Given that alcohol has served as a crucial tool for trust-building and group cohesion in almost all human cultures, the question arises as to why a trait that blocks access to this nearly universal social technology could prevail regionally.

Ecological models seek the selective advantage of an ALDH2 mutation in increased parasite resistance, as a sublethally elevated acetaldehyde level in the bloodstream – caused by the enzyme blockade – may have functioned as a chemical barrier against infections in humid rice fields (Oota et al., 2004). Current genomic analyses also emphasize the role of metabolic adaptation to a carbohydrate-rich rice diet (Landini et al., 2021). What these purely biological approaches overlook are the socio-structural opportunity costs of alcohol intolerance. In other regions with high parasite pressure, a comparable fixation of the mutation did not occur. Similarly, adaptation to other grain-based diets did not produce this effect. This suggests that in those regions, the social utility of alcohol continued to outweigh the biological costs of toxicity.

The present article attempts to close this gap by proposing a model of functional redundancy to explain alcohol intolerance. Rather than attributing the selection to biological drivers alone, this article adds an institutional economic perspective. It views functional redundancy as a facilitating socio-economic condition – one that lowered the social costs of alcohol intolerance while biological factors provided the selective pressure for its fixation. In a world where cooperation was objectively forced by the material infrastructure of rice cultivation, alcohol potentially lost its primary function as an instrument of social bonding. The mutation may therefore be interpreted as a potential biological correlate of an efficient social organization that arguably no longer required intoxication as a social glue.

## 2. The metabolic bottleneck

The human body typically processes alcohol in two successive steps. In the first step, ethanol is converted into acetaldehyde – a substance that is highly reactive, cytotoxic, and carcinogenic. Normally, an enzyme called ALDH2 ensures in the second step that this toxin is processed into harmless acetic acid before it can cause damage to the organism. In carriers of the ALDH2 mutation, this detoxification process is genetically blocked. The result is a toxic accumulation of acetaldehyde in the bloodstream that occurs even with small amounts of alcohol.

Those affected do not experience relaxation, but rather an immediate physical defence reaction: intense flushing (Asian Flush), tachycardia, nausea, and systemic inflammatory symptoms. Medically, this is primarily valued as a risk factor for carcinomas of the digestive tract (Brooks et al., 2009; Yokoyama et al., 2003). From an evolutionary perspective, it may be interpreted as a draconian chemical barrier that painfully punishes the consumption of alcohol. The fact that this barrier was not eliminated by selection suggests that the ability to consume alcohol may have ceased to offer a survival advantage in this specific environment.

## 3. Alcohol as social technology

In anthropological and economic literature, the consumption of alcohol is increasingly understood as a social technology that overcomes crucial bottlenecks in human cooperation. Humans face the evolutionary paradox of having to cooperate in anonymous large groups despite being biologically programmed for mistrust towards strangers. Slingerland (2021) describes alcohol as a tool for cognitive dampening. By inhibiting the prefrontal cortex – the part of the brain responsible for strategic planning, deception, and self-control – alcohol reduces the capacity for lying. Collective intoxication functions as a ‘hard-to-fake signal’: someone who is drunk is physically hardly capable of maintaining a complex, devious strategy. Alcohol thus created trust where no institutional guarantees existed.

Dunbar et al. (2017) supplement this approach with an endorphin hypothesis. Alcohol consumption stimulates the release of endorphins, which are essential for social bonding in groups. They view alcohol as a form of ‘social grooming’ that makes it easier to strengthen social ties in large groups than through physical interaction alone. Recent cross-cultural analyses by Hrnčíř et al. (2025) reinforce this premise, investigating whether alcohol consumption specifically facilitated the evolution of complex societies. These findings establish a global baseline: in most geographic and cultural contexts, chemical bonding agents were a primary pathway to overcoming the evolutionary challenges of large-group cooperation.

Douglas (1987) coined the term ‘constructive drinking’ for this: alcohol is used to construct social worlds and categories in the first place. Dietler and Hayden (2001) point to the economic function of drinking feasts. In many agrarian societies, alcohol serves as a medium to mobilize collective labour for projects that exceed the resources of an individual household, such as harvesting or clearing land. Here, alcohol transforms an economic necessity into a ritual social obligation.

In other words, alcohol was a significant lubricant for social stability and economic cooperation in most cultures. The opportunity costs of alcohol intolerance in these societies would have exposed carriers of the mutation to the risk of social marginalization. Why, then, was this different in the rice societies of East Asia?

## 4. Rain-fed versus irrigation-based agriculture

To understand the evolutionary path dependency of alcohol intolerance, one must analyse the material basis on which these societies emerged. An initial point of departure is Wittfogel’s (1957) concept of the ‘hydraulic society’, which argues that the form of agriculture determines the political and social structure of a civilization. For the present analysis, the critical distinction lies between rain-fed agriculture – such as wheat cultivation in Europe – and irrigation-based agriculture – such as paddy rice in Southern China. In rain-fed systems, production is largely individualistic; a farmer’s success depends on natural precipitation, and while cooperation with neighbours may be advantageous, it is not a strict prerequisite for daily operations.

In contrast, irrigation-based agriculture treats water as a scarce, flowing common-pool resource that must be managed through extensive systems of canals, dams, and reservoirs. In a rice society, cooperation is technically determined; an individual household cannot cultivate a rice field if the overarching system fails to function. This leads to significant social consequences, as the construction and maintenance of these channels require the disciplined, simultaneous labour of many individuals. This environment fostered early forms of management and bureaucratic planning, where social harmony became an economic necessity rather than merely a moral ideal. The individual is deeply integrated into the infrastructure, and those who violate norms risk exclusion from the water distribution system – a consequence with severe economic implications. Consequently, in rice-growing regions, structural trust is compelled by the physical environment, making social cohesion a product of material infrastructure.

## 5. The Rice Theory of Culture

Wittfogel’s sociological theses find modern empirical support in the research of Talhelm et al. (2014). Their ‘Rice Theory of Culture’ provides a psychometric foundation for the concept of functional redundancy proposed here. Using China as a case study, the authors provide evidence that historical cultivation methods (rice versus wheat) continue to shape the cognitive styles of the population today. Residents of historical rice-growing areas exhibit significantly more collectivist thinking patterns and a higher degree of social vigilance compared to those in wheat-growing regions. This psychological predisposition towards integration into collective structures represents the ‘software’ that complements the ‘hardware’ of the irrigation systems. In such an environment, the individual is embedded from birth in a network of obligations and mutual monitoring that leaves little room for deviant behaviour.

## 6. The narrative of functional redundancy

At this juncture, the socio-technological function of alcohol can be linked to the reality of the rice society. This leads to the central thesis of this paper: the functional redundancy of alcohol tolerance. While individualistic societies required alcohol to chemically lower barriers between autonomous individuals and artificially generate trust, this mechanism may have been redundant in a rice society. Cooperation was already secured by the material dependence on the water network. In these social fabrics, social control was so high that a ritual test of honesty via alcohol provided almost no additional information regarding a partner’s reliability; daily work on the canal was a far more valid signal.

The concept of functional redundancy, as applied here, refers to the phenomenon where a biological trait’s primary socio-economic utility is superseded by a more efficient institutional or material arrangement. In this framework, the scope of redundancy is specifically limited to ‘social technologies’, i.e., biological traits that facilitate group coordination. We can operationalize this as a shift in the cost–benefit ratio: when the marginal gain in trust provided by intoxication becomes negligible compared to the structural trust enforced by irrigation-based interdependencies, the metabolic cost of maintaining alcohol tolerance becomes an evolutionary liability.

In the logic of evolutionary economics, every biological trait carries costs. Maintaining alcohol tolerance requires energetic resources and carries risks of addiction and poor decision-making. Once the social function of alcohol became arguably superfluous due to the cooperation already enforced by rice cultivation, the cost–benefit ratio shifted dramatically. The ALDH2 mutation then emerged as an efficient protective measure, punishing the consumption of a toxin that offered no social surplus but caused physical harm. This suggests a scenario in which nature potentially phased out a technology as it was rendered redundant by superior social organization. The cultural choice for paddy rice cultivation created a social environment in which alcohol intolerance became a selective advantage, and once fixed, this genetic trait potentially reinforced a culture of sobriety and bureaucratic discipline, locking in the path of societal development for millennia.

## 7. Modern evidence: the Millwood study and the north–south divide

Theoretical derivations regarding functional redundancy are supported by findings in modern epidemiology and health services research. One of the most comprehensive investigations is the large-scale prospective cohort study by Millwood et al. (2019), which examined over 500,000 adults within the China Kadoorie Biobank. The Millwood study provides precise data on regional differences in alcohol consumption within China that extend beyond purely genetic explanations. In northern wheat regions, such as the provinces of Gansu or Henan, the proportion of regular male drinkers is significantly higher, and alcohol is both physically more present and culturally more embedded in social rituals. In southern rice regions, such as Sichuan or Zhejiang, the rate of abstainers is markedly higher.

The most critical result for the functional redundancy narrative, however, is not just the frequency of the mutation, but the intensity of consumption among those who do *not* carry it. Even individuals without the ALDH2 mutation tend to drink less in historical rice regions than their genetic counterparts in the north. This points to a cultural reinforcement: the sobriety historically necessitated by rice cultivation has created a cultural path that regulates consumption even where no biological barrier exists. The mutation historically acted as a biological catalyst for a development that today persists as a purely social institution, where cultural architecture has overlaid biological necessity.

## 8. Guanxi

The persistence of collective behavioural patterns documented in the Millwood study illustrates that the historical material infrastructure has left behind a profound social software. A central concept in Chinese sociology that illustrates this phenomenon is *Guanxi*. Analysis by Bian (2018) shows how this concept can be understood as a modern continuation of the material interdependence in rice societies. In the historical rice-growing areas of Southern China, *Guanxi* is structurally deep-seated, describing a system of mutual obligation that grew organically from the material dependence of the village community and kinship structures.

In contrast, in the wheat-growing regions of the north, *Guanxi* must often be laboriously built through ritual disinhibition and mutual proof of intent, frequently involving the use of alcohol. Here, intoxication serves as a necessary catalyst for trust between autonomous actors. The concept of functional redundancy may explain why the so-called Asian Flush is often accepted as an entirely legitimate signal in business contexts in the south. While refusing a toast (*Ganbei*) in the north may be viewed as a breach of trust-building, cooperation in the south is based more on long-term, materially grounded dependencies and networks of favours. The social bond here is so dense and secured by the legacy of collective infrastructure that the chemical detour via alcohol loses its economic advantage. *Guanxi* may therefore be viewed as a relational counterpart to historical irrigation-based cooperation, facilitating a degree of stability that persists independently of biological factors.

## 9. Genetic path dependency as evolutionary stabilization

In institutional economics, path dependency describes a process where early decisions – in this case, the choice of paddy rice cultivation – constrain the corridor for future developments. Rice production steered the society into a collectivist structure, which in turn rendered alcohol redundant as a bonding agent. This redundancy potentially provided an environment for evolution to fix a mutation that might otherwise have been eliminated elsewhere due to high opportunity costs, such as exclusion from social networks.

Once genetically fixed, alcohol intolerance likely further stabilized the cultural preference for sobriety, bureaucratic discipline, and implicit cooperation. This lock-in effect means that the biology of the population now serves to maintain the institutions of the past. The Asian Flush can therefore be conceptualized not as a defect, but as an evolutionary stabilizer of an efficient social niche.

## 10. Conclusion

The study of the ALDH2 mutation in East Asia reveals that evolutionary processes are steered not only by natural selection in the wild but significantly by the architecture of human social structures. The concept of functional redundancy provides a hypothetical explanation for a biological riddle: in the rice societies of East Asia, the ability to consume alcohol may have been lost not because it was inherently harmful, but because its primary social utility – the generation of trust and cohesion – had been more efficiently replaced by the material reality of rice cultivation.

It is important to emphasize that this institutional perspective is intended to be complementary to, rather than exclusive of, existing biological models. Alternative factors, as detailed in the Introduction, such as increased parasite resistance in humid paddy environments or metabolic adaptations to carbohydrate-heavy diets, likely provided the positive selective pressure required to fix the ALDH2 mutation. Functional redundancy likely acted as the enabling socio-economic condition that lowered the opportunity costs of losing alcohol tolerance, allowing these biological advantages to take hold without compromising social cohesion.

Furthermore, this model has limitations that warrant future interdisciplinary research. The ‘Rice Theory’ relies on historical agricultural data to infer past social behaviours, and the degree of genetic drift versus active selection remains a subject of ongoing genomic debate. While global research into alcohol traditions (Hrnčíř et al., 2025) suggests that chemical facilitators are often prerequisites for complex social organization, the historical rice regions of East Asia may provide a unique counter-example. In these societies, the material infrastructure of irrigation effectively rendered such chemical social technologies functionally redundant.

The example of alcohol intolerance in historical rice regions illustrates how human institutions, such as agricultural irrigation systems, may possess such power that they can influence the long-term trajectory of the biological hardware of our species. The genetic path dependency of alcohol intolerance may be viewed as an evolutionary strategy that potentially traded an expensive, risky social lubricant for a more stable and predictable method of cooperation rooted in bureaucratic discipline.

## Research transparency and reproducibility interest

The findings in the present paper do not rely on any data, code, or other resources.
